# A Point Prevalence Survey of Antimicrobial Use in Second-Level Mexican Hospitals: A Multicenter Study

**DOI:** 10.3390/antibiotics13111065

**Published:** 2024-11-09

**Authors:** German Alberto Venegas-Esquivel, María Guadalupe Berumen-Lechuga, Carlos José Molina-Pérez, Rodolfo Norberto Jimenez-Juarez, Enna Guadalupe Villanueva-Cabrera, David Vargas-González, Gonzalo Santos-González, Rebeca Pamela Velázquez Pérez, Mariana Hernández Navarrete, Celene Corral-Rico, Natali Robles-Ordoñez, Juan Manuel Lara-Hernández, Helen’s Irais Sánchez Mendoza

**Affiliations:** 1 Pediatric Department, Hospital de Ginecología y Obstetricia 221, Instituto Mexicano del Seguro Social (IMSS), Toluca 50000, Mexico; 2 Medical Research Coordination, Órgano de Operación Administrativa Desconcentrada Regional Mexico Poniente, Instituto Mexicano del Seguro Social (IMSS), Toluca 50000, Mexico; 3 Health Research Division, Unidad Médica de Alta Especialidad Hospitalde Ginecología y Obstetricia No. 4, Instituto Mexicano del Seguro Social (IMSS), Mexico 01090, Mexico; 4 Infectious Diseases Departament, Federico Gómez Children’s Hospital of Mexico, Mexico City 06720, Mexico; 5 Resident Emergency Departament, Hospital General Regional No. 251, Instituto Mexicano del Seguro Social (IMSS), Metepec 52148, Mexico; 6 Resident Emergency Departament, Hospital General de Zona número 194, Instituto Mexicano del Seguro Social (IMSS), El Molinito 53000, Mexico; 7 Resident General Surgery Department, Hospital General Regional 251, Instituto Mexicano del Seguro Social, Metepec 52104, Mexico; 8 Social Service Intern, Education and Research Deparment, Hospital General de Zona 252, Instituto Mexicano del Seguro Social (IMSS), Atlacomulco 50450, Mexico; 9 Neonatology Departament, Hospital para el Nino, Instituto Materno Infantil del Estado de Mexico, Toluca 50170, Mexico; 10 Infectious Diseases Department, Hospital para el Nino, Instituto Materno Infantil del Estado de Mexico, Toluca 50170, Mexico; 11 Intensive Care Department, Hospital de Ginecología y Obstetricia 221, Instituto Mexicano del Seguro Social (IMSS), Toluca 50000, Mexico; 12 Medical School, Autonomous University of the State of Mexico, Toluca 50180, Mexico

**Keywords:** point prevalence survey, antibiotic use, antimicrobial resistance, AWaRe antibiotic consumption, healthcare-associated infections, antimicrobial consumption

## Abstract

In 2018, the WHO published a methodology for conducting a point prevalence survey (PPS) of antibiotic use in hospitals. The aim of this study is to report the use of antibiotics in six second-level hospitals in Mexico using this methodology. Methods: A multicenter cross-sectional study based on the 2021–2023 adaptation for Latin American hospitals was conducted in internal medicine, surgery, intensive care unit (ICU), obstetrics and gynecology and pediatrics departments of the IMSS in the western region of the state of Mexico. Results: The overall prevalence of antibiotic use was 61%; the services with the highest prevalence of prescription were general surgery (79%) and the ICU (78%). A total of 846 patients were surveyed; there were no differences in antibiotic use or non-use in terms of gender, surgical procedure and invasive devices, but there were differences in median age and comorbidities. Adherence to guidelines was 53.9%. The three main antibiotics used were third-generation cephalosporins (28%), carbapenems (13%) and glycopeptides (9%); for the type of indication, for CAI and prophylaxis, the rates of use of third-generation cephalosporins were 29.2% and 44.5%, respectively, while for healthcare-associated infections, carbapenems were used (23.9%). By AWaRe group, the watch group was predominant for all types (63.9%), for prophylaxis it was the access group (39.3%), and for HAIs it was the reserve group (4.9%).

## 1. Introduction

Antimicrobial resistance (AMR) is one of the challenges in modern medicine which is considered a global public health problem, causing the increased morbidity and mortality of patients and the increased costs of hospital care [1,2,3]. AMR occurs when bacteria develop mechanisms to survive antibiotics, making the treatment of infectious diseases more challenging, and making other procedures such as surgery, chemotherapy, transplantation and others more risky or highly unsuccessful [1,3,4].

There are several factors that contribute to the evolution and spread of AMR like healthcare, agriculture, farming and the environment. These factors are strategic points for intervention at global and local levels, as declared in the global action plan published in 2015 by the World Health Organization (WHO) with five strategic objectives that favour the global awareness of and education and research on AMR, infection prevention and the optimal use of antibiotics to address the global problem [5,6].

One of the main issues in combating antibiotic resistance is the optimal use of antibiotics, because one of the main factors for the development of AMR is the excessive use and misuse of them. The research and development of new antibacterial agents has not been keeping up with the rate of evolution of antibiotic resistance, leaving a large breach in the ability to treat patients with infectious diseases. In 2024, priority interventions in the research and development of new antibacterials have been resumed to establish effective strategies to prevent, control and treat infections in relation to priority pathogens; these strategies, together with interventions directed by antimicrobial stewardship programmes (ASPs), favour the fight against AMR [6,7,8,9,10,11].

A global report in 2000 and 2015 established an increase in consumption based on defined daily doses of 65% (21.1–34.8 billion DDDs) and an increase in the consumption rate of 39% (11.3–15.7 DDDs per 1000 inhabitants per day); similarly, it has been projected that by 2030 consumption will be up to 200% higher if interventions to optimize antimicrobial use are not implemented [7,12,13].

Antibiotics are one of the most prescribed drugs; approximately 80% of their use takes place in the community, where the indiscriminate use of these drugs is observed. It is estimated that about 50% of these prescriptions are correct, although some studies have shown that this percentage may be as low as 30% in the community setting. Some reports estimate that about 55.9% of prescriptions are unsubstantiated; for pneumonia, treatment was validated in only 20% of cases and for urinary tract infections in 23.2%. These data reflect the urgent need to implement interventions to improve antimicrobial prescribing [14,15,16].

The knowledge on antibiotic prescription at both the hospital and community levels provides a situational diagnosis that will favour the interventions generated through ASPs at the local level and even some actions generated at the regional level and could lead to compliance with the global actions against AMR [6,17,18,19].

In 2018, the WHO published a methodology for conducting a point prevalence survey of antibiotic use (PPS) in hospitals, and in 2021–2023 an adaptation was made for Latin American countries; this methodology aims to collect information on antibiotic use in hospitals and can be adapted even to different countries or regions that favour knowledge of prescribing as part of active surveillance. These local situation diagnoses of antibiotic use can also provide a regional overview, thanks to assessments of different hospitals, and highlight the need to develop interventions against AMR through the actions of antimicrobial stewardship programmes [17,18,20].

The aim of this study is to evaluate the use of antibiotics in six second-level hospitals belonging to a regional Mexican health system, using the PPS methodology mentioned above.

## 2. Results

### 2.1. Characteristics of the Participating Hospitals

Six hospitals were included in the study, including a maternal perinatal specialty hospital (H6). A total of 846 patients were surveyed during the period from March to June 2024; the average number of patients screened per hospital was 106 (range 71–222), the departments with the highest number of participants were internal medicine, 35% (n = 295), and general surgery, 32% (n = 270), and the average hospital occupancy rate during the survey was 77.1% (range 54.6–84.7%) (Table 1).

### 2.2. Prevalence of Antibiotic Use by Hospitals and Attention Areas

The global prevalence of antibiotic use was 61%; the hospital with the most antibiotic use was H2 (66.8%) and H6 had the lowest prevalence (43.6%), the service with the highest prevalence of prescription was the general surgical department with 79%, followed by the intensive care unit with 78% (Table 2).

### 2.3. Clinical and Demographic Characteristics of the Study Population

There was no difference between groups in terms of gender, surgical procedure and invasive devices. In contrast, we found a difference in the median age between the groups, 46 vs. 51 years (antibiotic use vs. no antibiotic use), which is striking because a higher age would be expected in the antibiotic use group, but there were more newborns in this group, which contributed to the lower median age. We also found a difference in comorbidities overall (*p* 0.021); however, when we analyzed them by the type of the most common comorbidity, we found no differences (Table 3).

### 2.4. Microbiology Data

Microbiological reporting varied between hospitals; culture sampling for healthcare-associated infections (HAIs) and community-acquired infections (CAIs) was performed in 185 patients (47%), of which only 123 (66%) of the results were available at the time of the survey; 95 (67%) of the cultures were for HAIs and 90 (36%) were for CAIs (Supplementary Table S1). By the type of culture, 46 (25%) were blood cultures, 43 (26%) were urine cultures and 30 (16%) were bronchial secretion cultures (Supplementary Table S2).

Of the cultures with available results, 44/123 (36%) had a culture report without growth; 52/123 (42%) were ESKAPE group microorganisms (*E. faecium*, *S. aureus*, *K. pneumoniae*, *P. aeruginosa*, *E. coli*), and 26/123 (22%) were other microorganisms. In 20 (16%) cases, *E. coli* was reported as the most common microorganism (mainly in urine cultures), followed by *C. difficile* in 11 cases (9%) and *A. baumannii* in 10 (8.1%), mainly in bronchial secretion cultures.

The antimicrobial resistance approach by ESKAPE was variable by microorganism, however higher resistance was documented for *E coli* with MDR isolates in 18 cases (90%), for K pneumoniae 4 cases (66.6%) were considered MDR and for A baumannii 6 (60%) were XDR microorganisms (Supplementary Table S3).

### 2.5. Antibiotic Use

Of the 517 patients who received antibiotics, there were a total of 540 indications, of which 495 (91.7%) corresponded to 1 medical indication, 42 (7.8%) had 2 medical indications and only 3 (0.5%) had 3 indications for the use of antibiotics; 253 indications (47%) were related to CAIs, 141 (26%) to HAIs and 146 (27%) to prophylaxis. The most common community-acquired infection was pneumonia with 46 cases (18.1%), while ventilator-associated pneumonia, with 25 cases (17.7%), was the most common healthcare-associated infection (Supplementary Tables S4 and S5). In terms of prophylaxis, surgical prophylaxis was the most common with 136 patients (93.1%), of which 122 patients (89.7%) received multiple doses on more than one day (Supplementary Table S6).

Of the 540 indications, a total of 740 antibiotics were used, as each indication could have 2 to 3 antibiotics for the diagnosis; the 3 main antibiotics used for all indications were third-generation cephalosporins with a prevalence of 28% (n = 209), followed by carbapenems with 13% (n = 98) and finally glycopeptides with 9% (n = 70), which together represented 50% of the antibiotics used. When the use of antibiotics was divided according to the type of indication, third-generation cephalosporins were the most commonly used drugs for community-acquired infections with a prevalence of 29.2% (n = 107) and for prophylaxis with 44.5% (n = 77), whereas the use of carbapenems was more frequent for healthcare-associated infections with a prevalence of 23.9% (n = 48); the use of first-generation cephalosporins for prophylaxis alone was only 9.2% (n = 16) (Supplementary Table S7).

**Figure 1:**
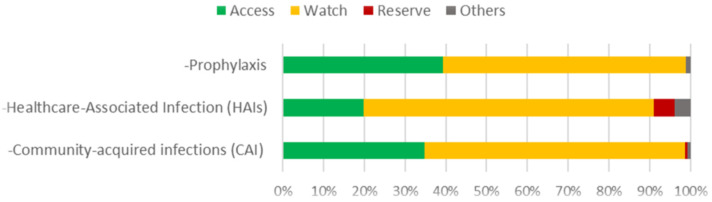
Antibiotic consumption by AWaRe group. Consumption was divided by type of indication.

For antibiotic prescribing by AWaRe group, the predominant indication in all types was the watch group, 63.9% for CAIs (n = 234), 71.1% for HAIs (n = 143) and 39.5% (n = 103) for prophylaxis, whereas the reserve group corresponded to 4.9% (n = 10) for HAIs, as shown in Figure 1.

### 2.6. Adherence to Guidelines of Antibotic Treatment by Department

Regarding adherence to guidelines, only 53.9% (n = 399) adhered to treatment guidelines; internal medicine was the service with the highest percentage of adherence to guidelines (33.1%). When comparing departments using the internal medicine service as a reference, only general surgery (50.1%) showed a clear difference with a *p* ˂ 0.001 (Table 4).

### 2.7. Functioning of Antimicrobial Sterwardship Programmes

A self-assessment of the performance of the antimicrobial stewardship programmes found a total performance of 40.7%, with the highest compliance in terms of clinical practice guidelines at 84.4%, and the lowest in the educational section at 17.9% (8.3–33.3). It is worth noting that, using unit H6 as a reference (which was the highest rated), we found a significant difference (*p* ˂ 0.001) when comparing continuous monitoring and surveillance specifically with unit H3, and when comparing H6 with the rest of the units in education and training (*p* ˂ 0.014) (Table 5).

## 3. Discussion

Point prevalence surveys allow for the identification of antibiotic use at the hospital level and provide a situation diagnosis that can promote actions for the better use of antibiotics, thus supporting the objectives of the Global Plan of Action against Antimicrobial Resistance [6,18,21].

In this multicenter cross-sectional study, a prevalence of antibiotic prescription of 61% was found, which is higher compared to other studies conducted in Europe and the United States; for example, a global report conducted in 2015, in which 53 countries participated, reported a prevalence of 34.4% in adult patients, although it should be noted that our study also included the pediatric population [22]. Another study conducted in the EU in 2015 with 199 hospitals documented a prevalence of 49.5% [23]. In China, a 2008 prevalence survey found 56% antibiotic use in general hospitals [24]. However, higher use has been reported in other developing countries in Asia and Africa; a study conducted in Pakistan [20,21,22] with 14 hospitals also found that the prevalence was higher when comparing government hospitals with private hospitals, 77% vs. 68%, while in Tanzania an even higher prevalence was reported (94.8%) [25,26]. However, a study that included different hospitals in the Latin American region reported an overall prevalence of 54%, highlighting Paraguay as the country with the highest prevalence of antibiotic use of 81.1%, while in Mexico the prevalence was 61.5% in the same study; another 2021 report in five hospitals in Mexico found a prevalence of antibiotic use of 66.3%, with both studies showing similar results to those found in our research [27,28]. Although the prevalence may vary by region and even by the individual characteristics of a hospital, as was the case in our H6 (maternal perinatal) with a frequency of antibiotic use of 43.6%, lower even than that reported in developed countries, the high prevalence of antibiotic use in the rest of the hospitals included in this study was consistent with local, multinational and inter-institutional reports [27,28].

Among the departments with the highest prescription rates, we found intensive care units had prevalence rates of up to 100% (67–100) and general surgery departments had an average of 79%, higher than those reported in prevalence studies worldwide and even in countries in the Americas [22,23,24,27]; compared to local data from Mexico, the prevalence in these departments is even higher than reported [27,28], but similar to a study from Vietnam in 2012 where the prevalence in surgical departments was even higher [29], reflecting the need to intervene in surgical and intensive care departments where antibiotic abuse is more prevalent.

Regarding the taking of cultures, the frequency was 47% (n = 185), and only 123 (66%) were available at the time of evaluation, similar to that reported in Latin America (44.3%) but lower than that reported in Mexico (59.2%); this lack of taking cultures is the reason why targeted antibiotic treatment is avoided, finding only 9% targeted therapy in community infections and 39% in healthcare-associated infections in our study; this differs from data reported in global studies (19.8%) and Latin America (17.2%) regarding the targeted use of antibiotics, reflecting the need for training in the appropriate culture collection to provide a result that allows for targeted antibiotic therapy, avoiding or decreasing the use of broad-spectrum antibiotics and supporting the adjunctive actions of ASPs [30,31,32,33].

The antibiotic resistance in the reports available at the time of evaluation is consistent with other reports in Mexico: the resistance of *E. coli* to third-generation cephalosporins is estimated at 50–60%, *S. aureus* MRSA 30–40%, *A. baumannii* resistant to carbapenems at >80% and *K. pneumoniae* resistant to third-generation cephalosporins at 50–60% [2]. While other local reports in Mexico showed resistance to third-generation cephalosporin by *E. coli* in 40–70% of cases, third-generation cephalosporin-resistant *K. pneumoniae* in 30–70%, carbapenem-resistant *P. aeruginosa* in 20–40% and carbapenem-resistant *A. baumannii* in 40–80%; regarding resistance patterns, *E. coli* MDR occurred in 53.4–62.9% of cases, *K. pneumoniae* MDR in 44.6% to 50.5%, *A. baumannii* possible XDR in 60% to 78.2% and *P. aeruginosa* possible XDR in 19.5% to 27.1% [34]. Resistance patterns were documented at the Latin American scale through the presence of carbapenemases, with reports of *A. baumannii* resistance of 21% to 89% and resistance for *P. aeruginosa* of 14% to 64.6% [35]. In a regional study conducted in one of the hospitals evaluated (H1), *E. coli* resistance to third-generation cephalosporins was reported at 76%, *K. pneumoniae* resistance to third-generation cephalosporins at 69%, carbapenem resistance in *A. baumannii* at higher than 92% and in *P. aeruginosa* at 57% and *S. aureus* MRSA at 27% [36]. The results of our study compared to these studies are similar; however, the number of isolates is too limited to be able to generalize the antimicrobial resistance pattern. Nevertheless, the resistance patterns identified reflect the need for broad-spectrum antibiotic treatment regimens; ASP interventions should aim for optimal diagnostic and treatment stewardship to improve the outcome of both empiric and targeted therapies [11,19,33,37,38].

In relation to surgical prophylaxis, the prevalence reported in the Americas and other regions is <30% of prescriptions; continuing with the multinational study of hospitals in Latin America, Mexico had a prescription rate of 11.6% to 25% [22,27,28], so when compared with our findings (27%), the result is similar. Surgical prophylaxis is usually based on the use of first-generation cephalosporins as a single dose prior to surgery [39,40]; however, worldwide we see different regimens. For example, in a prevalence study in Ethiopia, adherence to single doses was only 3.2% [41], whereas in the Americas adherence to guidelines was reported at 44.3%; however, Mexico was the country with the lowest reported adherence at 28.5%. Similarly, in a study of Mexican hospitals, the frequency of single-dose prophylaxis ranged from 1.9% to 22.2%, which is comparable to our results, where the average frequency of single-dose prophylaxis was 10% (0–26%) and only 22% of these prescriptions adhered to practice guidelines. The use of first-generation cephalosporins for prophylaxis in Latin America averaged 35.7%; however, in Mexico, third-generation cephalosporins were the most commonly used (40.4%), which is consistent with our research, since the frequency of third-generation cephalosporins indicated for surgical prophylaxis was 45% [27,28]. The above reflects the abuse of antibiotics not only at a regional level, but also at a global level in surgical departments, which represents a great opportunity to optimize the use of antimicrobials. The global prescribing rates in Latin America vs. Mexico for community infections are comparable, since about half of the prescriptions are based on the use of third-generation cephalosporins (30.6% vs. 30.4%), quinolones (9.7% vs. 10.1%) and carbapenems in 8.4% vs. 13.2% of cases; in hospital infections, the most prescribed antibiotics in Latin America are carbapenems at 21.4% and vancomycin at 16.3%, compared to carbapenems at 22.1%, glycopeptides at 20.1% and third-generation cephalosporins at 7.6% in Mexico [27]. In a worldwide study, the use of third- and fourth-generation cephalosporins was reported to be 14.4% [23].

Antibiotic consumption by AWaRe group is reported as 57.7% for the watch group, 40% for access and 0.4% for reserve; for prophylaxis, the highest consumption is for the access group at 57.9% and the watch group at 60.1%, while for community and hospital infections it is 64.8% [27]. The GLASS 2022 report estimates an average antibiotic consumption of 67% for the access group, 31% for the watch group and 0.15% for the reserve group; however, Mexico does not report to this international system [42]. In Mexico, a report from the Universidad Nacional Autónoma de México 2022 reported a higher consumption of third-generation cephalosporins at 27 DDD/100 beds and carbapenems at 7 DDD/100 beds [43]. A local report reported the consumption of drugs from the access group as 35.3%, vigilance as 63.87% and reserve as 0.77%, with cephalosporins being the most consumed [36]. In terms of correct prescribing, a global study reported an adherence to treatment guidelines of 77.4% and 68.6% for Latin America, while Mexico reported an adherence of 72.6%; our results showed a significantly lower adherence of 53.8% and are far from the international AWaRe target for consumption by group (60% of antibiotics in the access group); this target refers to global consumption and does not differentiate between hospital and community infections, in addition to the increase in AMR found in the study hospitals. Nevertheless, these results highlight the inappropriate use of these drugs and reinforce the need for national and local interventions to improve the prescription of antibiotics in hospital units through the ASP.

For the WHO self-assessment of antimicrobial stewardship programmes, a performance of 40.7% (17.9–84.4%) was achieved at the global, delegational level. After comparisons between groups and taking unit H6 as the reference (as it achieved the best performance), there were no differences in administrative support for the ASP and infrastructure team, clinical practice guidelines and antimicrobial prescribing strategies, but there were differences in the aspects of ongoing monitoring and surveillance and education and training (*p* 0.014), indicating an opportunity to direct efforts towards strengthening these areas, as well as antimicrobial prescribing strategies, which represent the three areas with the lowest performance globally and by unit.

One of the limitations of our study is the generalization of the results, as each hospital has its own characteristics and a different structure. However, a large number of prescriptions were analyzed in the same region and in the same institution in a low–middle income country. We consider it a strength that the study sample was obtained through specific training in the correct application of the survey, accompanied by constant communication and review, which gave consistency to the data obtained and allowed us to identify the local and regional problems regarding the high percentage of the inappropriate use of antibiotic prescriptions and non-adherence to guidelines.

## 4. Materials and Methods

We conducted a multicentre cross-sectional study at the Instituto Mexicano del Seguro Social, a network of six hospitals serving more than 2.5 million people in the western region of the state of Mexico.

### 4.1. Hospital Description

Six hospitals were included in the study; these hospitals are part of a delegation that provides care to the western part of the state of Mexico, with one maternal perinatal specialty hospital and the five other hospitals providing general second-level care, represented as H1 (Hospital General Regional 251, Metepec, Mexico, 262 beds), H2 (General Regional Hospital 220, Toluca, Mexico, 257 beds), H3 (Hospital General de Zone 194, Naucalpan, Mexico, 219 beds), H4 (Hospital General de Zone 58, Tlalnepantla, Mexico, 130 beds), H5 (Hospital General de Zone 252 (H5), Atlacomulco, 98) and H6 (Hospital of Obstetrics and Gynaecology 221, Toluca, Mexico, 130 beds). During the study period, hospitals H1 and H3 underwent the remodelling of wards with a reduction of about 30 beds; however, the units continue to provide the usual hospital care, mainly in affected internal medicine departments.

### 4.2. Data Collection

The PPS was conducted based on the methodology described by the WHO and its 2021–2023 adaptation for Latin American hospitals; the survey’s duration was no longer than 3 weeks per hospital. All hospitalized patients were screened according to the daily record of the hospital department at 8:00 am, regardless of whether they had received antibiotic treatment or not; outpatient services were excluded, such as emergency, haemodialysis and ambulatory peritoneal dialysis [44].

To develop the survey, all patients in a particular department were assessed on a single day, once during the study period. The hospital departments were divided into internal medicine, surgery, the adult intensive care unit (AICU), obstetrics and gynecology and pediatrics.

The assessment of prophylaxis, infectious diseases and adherence to treatment guidelines was based on internal guidelines, based on hospital microbiology, international guidelines and the supervision of the PROA teams; if no internal guidelines were available, clinical practice guidelines (CPGs) were used, and if no guidelines were available, they were considered not evaluable.

Isolates were classified as multi-drug-resistant (MDR) when non-susceptibility to at least one antibiotic in the three antimicrobial classes tested was documented, extensively drug-resistant (XDR) when non-susceptibility to at least one antibiotic in all but two or fewer antimicrobial classes was reported and pan-drug-resistant (PDR) when non-susceptibility to all antibiotics in all antimicrobial classes tested was documented [44].

The sample was collected by two staff members of each study unit, who completed the electronic form and were trained by an infectious disease specialist. A self-assessment tool developed by the WHO was used to evaluate the performance of the ASP; this evaluation was completed by the ASP teams of each unit.

### 4.3. Statistical Analysis

Categorical variables were presented as frequencies and percentages, while continuous variables were presented as medians and interquartile ranges as they do not have a normal distribution. The ratio of the number of patients with antimicrobial use to the number of patients included was used to determine the prevalence of antimicrobial use.

The chi-squared test was used for categorical variables and the Mann–Whitney U test for continuous variables to compare groups’ antibiotic use or non-antibiotic use, adherence or non-adherence to guidelines, and the WHO self-assessment of antimicrobial stewardship programmes per unit. SPSS version 27 statistical software was used for data analysis.

## 5. Conclusions

The overall prevalence of antibiotic use was 61%; the services with the highest prevalence of prescription were general surgery (79%) and the ICU (78%). The adherence to guidelines was only 53.9%. Our results reinforce the need for actions to combat AMR based on education, awareness about the rational use of antibiotics and the importance of infection prevention and control, but mainly for the development of national programmes that can take on a life of their own in each of the healthcare units. Support from the authorities and protected time is essential for the development of ASPs to support correct prescribing and tackle AMR.

## Figures and Tables

**Table 1:** Characteristics of six hospitals and occupation rate.

Hospitalization Departments *N* = 846	H1 *n* = 222	H2 *n =* 202	H3 *n =* 169	H4 *n =* 101	H5 *n =* 81	H6 *n =* 71
Total Hospital Occupation, (%)	84.7	78.5	77.1	77.6	82.6	54.6
**Patients Evaluated by Department**						
General Surgical, n (%)	80 (36.0)	81 (40.1)	55 (32.5)	26 (25.7)	28 (34.6)	N/A
Internal Medicine, n (%)	54 (24.3)	85 (42.1)	69 (40.8)	54 (53.4)	33 (40.7)	N/A
Obstetrics and Gynecology, n (%)	45 (20.2)	N/A	31 (36.1)	N/A	8 (9.9)	52 (73.2)
Pediatric, n (%) *	36 (16.2)	31 (15.3)	12 (7.1)	19 (18.8)	8 (9.8)	16 (22.5)
AICU, n (%)	7 (3.1)	5 (2.5)	2 (1.2)	2 (2.0)	4 (4.9)	3 (4.2)

Data are expressed as frequencies and percentages (n, %). N/A (Not Applicable, service not available), AICU (Adult Intensive Care Unit). * Including neonatal intensive and intermediate care units and departments such as maternal co-housing and neonatal growth.

**Table 2:** Prevalence of antibiotic use by hospitals and attention areas.

Ward Prevalence	H1 *n =* 222	H2 *n =* 202	H3 *n =* 169	H4 *n =* 101	H5 *n =* 81	H6 *n =* 71	Global *N =* 846
Antibiotic Use Prevalence, n (%)	146 (65.7)	135 (66.8)	99 (58.6)	59 (58.4)	47 (58.0)	31 (43.6)	517 (61.1)
**Hospital Wards**							
General Surgical, n (%)	65 (81)	63 (78)	45 (82)	21 (81)	20 (71)	N/A	214 (79)
Internal Medicine, n (%)	34 (63)	48 (56)	32 (46)	25 (46)	15 (45)	N/A	154 (52)
Obstetrics and Gynecology, n (%)	20 (44)	N/A	15 (48)	N/A	3 (38)	24 (46)	62 (46)
Pediatric, n (%) *	22 (73)	20 (76)	5 (41.5)	11 (58)	5 (56)	5 (39.0)	68 (69)
AICU, n (%)	5 (71)	4 (80)	2 (100)	2 (100)	3 (75)	2 (67)	18 (78)

Data are expressed as frequencies and percentages (n, %). N/A (Not Applicable, service not available), AICU (Adult Intensive Care Unit). * Including neonatal intensive and intermediate care units and departments such as maternal co-housing and neonatal growth.

**Table 3:** Clinical and demographic characteristics of the study population.

Variable *N* = 846	Antibiotic Use *n* = 517	Non-Antibiotic Use *n* = 329	*p* Value
Gender			0.331 *
Female, n [%]	256 (49.5)	175 (53.2)	
Male, n [%]	261 (50.5)	154 (46.8)	
Age, years, median [IQR]	46 (21–63)	51 (26–68)	0.006 **
Comorbidities ǂ	348 (67.3)	195 (59.2)	0.021 *
Absence of comorbidity, n [%]	169 (32.7)	134 (40.7)	Reference
Chronic hypertension, n [%]	178 (32.9)	122 (37.1)	0.603 *
Diabetes, n [%]	148 (28.6)	97 (29.5)	0.314 *
Chronic kidney disease, n [%]	91 (17.0)	45 (15.6)	0.036 *
Others, n [%]	28 (5.42)	19 (5.78)	0.94 *
Surgical procedure, n [%]	175 (33.8)	110 (33.4)	0.960 *
Nutritional state			
Normal weight, n [%]	202 (39.1)	157 (50.7)	Reference
Malnutrition, n [%]	93 (18.0)	26 (7.9)	˂0.001
Overweight, n [%]	119 (23.0)	96 (29.2)	0.320
Obesity, n [%]	103 (19.9)	50 (15.2)	0.025
Invasive devices ǂ			
Peripheric vascular catheter, n [%]	402 (77.7)	242 (73.5)	0.118 *
Urinary catheter, n [%]	128 (24.7)	77 (23.4)	0.714 *
Central vascular catheter, n [%]	83 (16.0)	46 (14.0)	0.471 *
Peritoneal catheter	24 (4.6)	12 (3.6)	0.600 *
Haemodialysis catheter, n [%]	27 (5.2)	16 (4.9)	0.943 *
Orotracheal intubation, n [%]	60 (11.6)	27 (8.20)	0.141 *

Data are presented as count and frequency n [%] and IQR, interquartile range. A *p* value of *p* < 0.05 was considered statistically significant. * Chi-squared test, ** U Mann–Whitney. ǂ The total is higher because the patient had two or more comorbidities and two or more catheters.

**Table 4:** Adherence to treatment guidelines by department.

Variable *N* = 740	Adherence to Guidelines *n* = 399	Non-Adherence to Guidelines *n* = 341	*p* Value
Internal medicine, n (%)	132 (33.1)	77 (22.6)	Reference
General surgical, n (%)	129 (32.3)	171 (50.1)	˂0.001
Pediatrics, n (%)	79 (19.8)	37 (10.9)	0.439
Gynecology and obstetrics, n (%)	43 (10.8)	43 (12.6)	0.049
Adult intensive care unit, n (%)	16 (4.0)	13 (3.8)	0.530

Data are expressed as frequencies and percentages; three cases were excluded because adherence was not evaluable (two in general surgery and one in internal medicine).

**Table 5:** WHO self-assessment of antimicrobial stewardship programmes.

Variable	Delegational Result	H1	H2	H3	H4	H5	H6 Reference	*p* Value
Administrative support, for ASP and infrastructure team (%)	43.7	41	39.1	37.3	37.9	51.2	50.7	NS
Clinical practice guidelines	84.4	83.3	86.1	83.3	86.1	83.3	83.3	NS
Antimicrobial prescribing strategies	28.8	28.1	33.3	25.0	25.0	25.0	36.5	NS
Ongoing monitoring and surveillance	27.5	28.6	30.2	15.6	24.5	24.0	42.2	˂0.001 *
Education and training	17.9	16.7	16.7	8.3	16.7	16.7	33.3	0.014 ** ˂0.001 *
Global delegational result	40.7	39.5	40.5	33.9	37.9	40.0	49.2	NS

* H6vsH3, ** H6vsH1, H2, H4, H5.

## Data Availability

Data that support the findings of this study are available from the corresponding author upon reasonable request.

## Supplementary Materials

The following supporting information can be downloaded at https://www.mdpi.com/article/10.3390/antibiotics13111065/s1: Table S1, Supplementary Material: Microbiological culture samples collected, Table S2, Supplementary Material: Types of microbiological culture samples taken; Table S3, Supplementary Material: Antimicrobial resistance profile; Table S4, Supplementary Material: Antibiotic indication in CAIs; Table S5, Supplementary Material: Antibiotic indications in HAIs; Table S6, Supplementary Material: Antibiotic indication in prophylaxis indications; Table S7, Supplementary Material: Antibiotic use for different indications.
