# Supplementary material for: A Point Prevalence Survey of Antimicrobial Use in Second-Level Mexican Hospitals: A Multicenter Study
Source: Antibiotics (Basel). 2024 Nov 9;13(11):1065. doi: 10.3390/antibiotics13111065 (PMC11591313; doi:10.3390/antibiotics13111065)
Supplement: Supplementary file 1 [file antibiotics-13-01065-s001.zip › antibiotics-3251902-supplementary.pdf]

**Table S1.** Supplementary material. Microbiological culture samples collected.

| Variable | H1 *n=47* | H2 *n=67* | H3 *n=13* | H4 *n=29* | H5 *n=24* | H6 *n=5* |
|---|---|---|---|---|---|---|
| Available results, n (%) | 29 (61.7) | 47 (70.1) | 8 (61.5) | 22 (75.8) | 16 (66.6) | 1 (20) |
| **Type of cultures** | | | | | | |
| CAIs culture sample, n (%) | 30 (63.8) | 24 (35.8) | 7 (53.8) | 8 (36.3) | 16 (66.6) | 5 (100) |
| HAIs culture sample, n (%) | 17 (36.1) | 43 (64.1) | 6 (46.1) | 21 (72.4) | 8 (33.3) | 0 (0) |

Data are expressed as frequencies and percentages (n, %). N/A (Not Applicable, service not available).

**Table S2.** Supplementary material. Types of microbiological culture samples taken.

| Type of microbiological culture (N) | H1 *n=47* | H2 *n=67* | H3 *n=13* | H4 *n=29* | H5 *n=24* | H6 *n=5* |
|---|---|---|---|---|---|---|
| Blood culture, n (%) | 6 (12.7) | 16 (23.8) | 3 (23) | 12 (41.3) | 6 (25) | 3 (60) |
| Urine culture, n (%) | 19(40.4) | 7(10.4) | 2 (15.3) | 5 (17.2) | 9 (37.5) | 1 (20) |
| Bronquial culture, n (%) | 10 (21.2) | 10 (14.9) | 5 (38.4) | 4 (13.7) | 1 (4.17) | N/A |
| Sterile fluids (CSF, Peritoneal Synovial, etc.), n (%) | 7 (14.8) | 13 (19.4) | N/A | 4 (13.7) | 4 (16.7) | N/A |
| Wounds/Secretions, n (%) | 5 (10.6) | 9 (13.4) | 2(15.3) | 4 (13.7) | 4 (16.7) | 1 (20) |
| Stool Samples, n (%) | N/A | 11 (16.4) | 1 (7.69) | N/A | N/A | N/A |
| Catheter tip, n (%) | N/A | 1(1.4) | N/A | N/A | N/A | N/A |

Data are expressed as frequencies and percentages (n, %). N/A (Not Applicable, service not available).

**Table S3.** Supplementary material. Antimicrobial Resistance Profile.

| Microorganism | Total | Unknown | Susceptible | MDR | XDR |
|---|---|---|---|---|---|
| *Enterococcus spp*, n (%) | 5 | 1 (20) | 3 (60) | 1 (20) | N/A |
| *Staphylococcus aureus*, n (%) | 6 | 1 (16.6) | 4 (66.6) | 1 (16.6) | N/A |
| *Klebsiella pneumoniae*, n (%) | 6 | 1(16.6) | N/A | 4 (66.6) | 1 (16.6) |
| *Acinetobacter spp*, n (%) | 10 | 2 (20) | 2 (20) | N/A | 6 (60) |
| *Pseudomonas aeruginosa*, n (%) | 5 | 1 (20) | 2 (40) | N/A | 2 (40) |
| *Escherichia coli*, n (%) | 20 | N/A | 1 (5) | 18 (90) | 1 (5) |

Data are expressed as frequencies and percentages (n, %). N/A (Not Applicable, service not available), multi-drugresistant (MDR), extensively drug-resistant (XDR).

**Table S4.** Supplementary material. Antibiotic indications in CAIs.

| Community acquired infections (CAIs) | H1 *n=74* | H2 *n=53* | H3 *n=56* | H4 *n=30* | H5 *n=28* | H6 *n=12* | Global *N=253* |
|---|---|---|---|---|---|---|---|
| **Therapy provided** | | | | | | | |
| Targeted therapy, n (%) | 10 (14) | 6 (11) | N/A | 2 (7) | 5 (18) | N/A | 23 (9) |
| Empiric therapy, n (%) | 64 (86) | 47 (89) | 56 (100) | 28 (93) | 23 (82) | 12 (100) | 230 (91) |
| **Diagnostic indications for community acquired** | | | | | | | |
| Pneumonia, n (%) | 13 (18) | 10 (19) | 10 (18) | 9 (30) | 4 (14) | N/A | 46 (18) |
| Cellulitis, wound, deep soft tissue without bone involvement, not associated with surgery, n (%) | 12 (16) | 14 (26) | 11 (20) | 5 (17) | 2 (7) | 1 (8) | 45 (18) |
| Symptomatic infection of superior urinary tract (pyelonephritis), n (%) | 15 (21) | 6 (11) | 10 (18) | 2 (7) | 8 (27) | 1 (8) | 42(17) |
| Intrabdominal sepsis, also hepatobiliary, n (%) | 9 (12) | 8 (15) | 13 (23) | 4 (14) | 3 (11) | N/A | 37 (15) |
| Clinical sepsis: suspected bloodstream infection without laboratory confirmation / results not available / hematic culture absent or without result, excluding febrile neutropenia, n (%) | 13 (18) | 3 (6) | 1 (2) | 1 (3) | 3 (11) | 6 (50) | 27 (11) |

| | | | | | | | |
|---|---|---|---|---|---|---|---|
| Dialysis-associated peritonitis, n (%) | 1 (1) | 6 (11) | 2 (3) | 1 (3) | 3 (11) | N/A | 13 (5) |
| Symptomatic infection of inferior urinary tract (cystitis), n (%) | 1 (1) | 1 (2) | 2 (3) | 3 (10) | 1 (4) | 3 (26) | 11 (4) |
| Central Nervous System Infections, n (%) | 3 (4) | 3 (6) | 1 (2) | 1 (3) | N/A | N/A | 8 (3) |
| Obstetric or gynaecological infections, ITS in women, n (%) | 1 (1) | N/A | 5 (9) | N/A | N/A | 1 (8) | 7 (2) |
| Febrile neutropenia or some other form of immunosuppressed host infection without clear anatomic site, n (%) | 4 (6) | N/A | N/A | N/A | N/A | N/A | 4 (1) |
| Septic arthritis, surgical site osteomyelitis, n (%) | N/A | 1(2) | N/A | N/A | 2 (7) | N/A | 3 (1) |
| Gastrointestinal infections: salmonellosis, diarrhoea associated to antibiotics, n (%) | N/A | N/A | N/A | 2 (7) | 1 (4) | N/A | 3 (1) |
| Infections of the nose, throat, ear, larynx and mouth, n (%) | 1 (1) | N/A | N/A | 1 (3) | 1 (4) | N/A | 3 (1) |
| Other, n (%) | 1 (1) | 1 (2) | N/A | N/A | N/A | N/A | 2 (1) |
| Asymptomatic bacteriuria, n (%) | N/A | N/A | 1 (2) | N/A | N/A | N/A | 1 (1) |
| Prostatitis, epididymis-orchitis, STDs in men, n (%) | N/A | N/A | N/A | 1 (3) | N/A | N/A | 1 (1) |

Data are expressed as frequencies and percentages (n, %). N/A (Not Applicable, service not available).

**Table S5.** Supplementary material. Antibiotic indications in HAIs.

| **Hospital Associated Infections (HAI)** | **H1** *n=28* | **H2** *n=55* | **H3** *n=22* | **H4** *n= 25* | **H5** *n=11* | **H6** *N/A* | **Global** *N=141* |
|---|---|---|---|---|---|---|---|
| **Therapy** | | | | | | | |
| Targeted therapy, n (%) | 10 (36) | 29 (53) | 5 (23) | 9 (36) | 2 (18) | N/A | 55 (39) |
| Empiric therapy, n (%) | 18 (64) | 26 (47) | 17 (77) | 16 (64) | 9 (82) | N/A | 86 (61) |
| **Diagnostic indications for healthcare associated infections** | | | | | | | |
| Mechanical ventilation associated Pneumonia, n (%) | 6 (22) | 10 (18) | 4 (18) | 1 (4) | 4 (37) | N/A | 25 (18) |
| Pneumonia, n (%) | 7 (25) | 4 (7) | 5 (23) | 4 (16) | 2 (18) | N/A | 22 (16) |
| Surgical site infection with dermis or soft tissue involvement, without bone involvement, n (%) | 3 (11) | 8 (15) | 4 (18) | 3 (12) | 2 (18) | N/A | 20 (14) |
| Catheter related infection, n (%) | 3 (11) | 7 (13) | 2 (9) | 4 (16) | N/A | N/A | 16 (11) |
| Gastrointestinal infections: salmonellosis, diarrhoea associated to antibiotics, n (%) | 1 (3) | 12 (22) | 1 (5) | N/A | N/A | N/A | 14 (10) |
| Symptomatic infection of superior urinary tract (pyelonephritis), n (%) | 3 (11) | 2 (4) | 2 (9) | 3 (12) | N/A | N/A | 10 (7) |
| Clinical sepsis: suspected bloodstream infection without laboratory confirmation / results not available / hematic culture absent or without result, excluding febrile neutropenia, n (%) | N/A | 3 (5) | 4 (18) | 1 (4) | N/A | N/A | 8 (6) |
| Intrabdominal sepsis, also hepatobiliary, n (%) | N/A | 3 (5) | N/A | 4 (16) | N/A | N/A | 7 (5) |
| Dialysis-associated peritonitis, n (%) | 3 (11) | 2 (4) | N/A | 1 (4) | 1 (9) | N/A | 7 (5) |
| Symptomatic infection of inferior urinary tract (cystitis), n (%) | N/A | N/A | N/A | 2 (8) | 2 (18) | N/A | 4 (3) |
| Laboratory confirmed bacteraemia, n (%) | N/A | 3 (5) | N/A | 1 (4) | N/A | N/A | 4 (3) |
| Cellulitis, wound, deep soft tissue without bone involvement, not associated with surgery, n (%) | 1 (3) | N/A | N/A | 1 (4) | N/A | N/A | 2 (1) |
| Septic arthritis, surgical site osteomyelitis, n (%) | 1 (3) | 1 (2) | N/A | N/A | N/A | N/A | 2 (1) |

Data are expressed as frequencies and percentages (n, %). N/A (Not Applicable, service not available).

**Table S6.** Supplementary material. Antibiotic indication in prophylaxis indications.

| Prophylaxis indications | H1 *n=48* | H2 *n= 34* | H3 *n=25* | H4 *n=5* | H5 *n=14* | H6 *n=19* | Total *N=146* |
|---|---|---|---|---|---|---|---|
| **Prophylaxis type** | | | | | | | |
| Medical prophylaxis, n (%) | 4 (8) | 2 (6) | 1 (4) | 1 (20) | 2 (14) | N/A | 10 (7) |
| Surgical prophylaxis, n (%) | 44 (94) | 32 (94) | 25 (96) | 4 (80) | 12 (86) | 19 (100) | 136 (93) |
| **Surgical prophylaxis duration** | | | | | | | |
| One Dose, n (%) | 6 (14) | 1 (3) | N/A | N/A | 2 (17) | 5 (26) | 14 (10) |
| Multiple doses more than one day, n (%) | 38 (86) | 31 (97) | 25 (100) | 4 (100) | 1 (83) | 14 (74) | 122 (90) |

Data are expressed as frequencies and percentages (n, %). N/A (Not Applicable, service not available).

**Table S7.** Supplementary material. Antibiotic use for different indications.

| ATC Code/Antibiotic name | Community-acquired infections (CAI) n= 366 | Healthcare-Associated Infection (HAIs) n=201 | Prophylaxis n= 173 | Global N=740 |
|---|---|---|---|---|
| **J01DD Third-generation cephalosporins, n (%)** | | | | |
| Cefotaxime | 34(9.3) | 6(3) | 22(12.7) | 62 (8.4) |
| Ceftazidime | 8 (2.2) | 8(4) | 0(0) | 16 (2.2) |
| Ceftriaxone | 65(17.8) | 11(5.5) | 55(31.8) | 131(17.7) |
| **J01DH Carbapenems, n (%)** | | | | |
| Ertapenem | 3(0.8) | 2(1) | 0(0) | 5(0.7) |
| Imipenem, cilastatin | 3(0.8) | 8(4) | 0(0) | 11(1.5) |
| Meropenem | 43(11,7) | 38(18.9) | 1(0.6) | 82(11.1) |
| **J01XA Glycopeptide antibacterials, n (%)** | | | | |
| Vancomycin | 26(7.1) | 42(20.9) | 2(1.2) | 70(9.5) |
| **J01XD Imidazole derivatives, n (%)** | | | | |
| Metronidazole | 35(9.6) | 14(7) | 16(9.2) | 65(8.8) |
| **J01MA Fluoroquinolones, n (%)** | | | | |
| Ciprofloxacin | 7(1.9) | 3(1.5) | 9(5.2) | 19(2.6) |
| Levofloxacin | 25(6.8) | 14(7) | 6(3.5) | 45(6.1) |
| Moxifloxacin | 1(0.3) | 0(0) | 0(0) | 1(0.1) |
| **J01FF Lincosamides, n (%)** | | | | |
| Clindamycin | 28(7.7) | 8(4) | 14(8.1) | 50(6.8) |
| **J01GB Other aminoglycosides, n (%)** | | | | |
| Amikacin | 25(6.8) | 7(3.5) | 8(4.6) | 40(5.4) |
| Gentamicin | 1(0.3) | 1(0.5) | 0(0) | 2(0.3) |
| **J01CA Penicillin with extended spectrum, n (%)** | | | | |
| Amoxicillin | 0(0) | 0(0) | 4(2.3) | 4(0.5) |
| Ampicillin | 16(4.4) | 1(0.5) | 6(3.5) | 23(3.1) |
| **J01DB First-generation cephalosporins, n (%)** | | | | |
| Cefalotin | 1(0.3) | 0(0) | 16(9.2) | 17(2.3) |
| **J01CR Combinations of penicillin, including beta-lactamase inhibitor, n (%)** | | | | |
| Amoxicillin and beta-lactamase inhibitor | 1(0.3) | 1(0.5) | 0(0) | 2(0.3) |
| Piperacillin and beta-lactamase inhibitor | 9(2.5) | 3(1.5) | 0(0) | 12(1.6) |
| **J01DE Fourth-generation cephalosporins, n (%)** | | | | |
| Cefepime | 8(2.2) | 5(2.5) | 0(0) | 13(1.8) |
| **J01FA Macrolides, n (%)** | | | | |
| Clarithromycin | 10(2.7) | 2(1) | 0(0) | 12(1.6) |
| **J02AC Triazole and tetrazole derivatives, n (%)** | | | | |
| Fluconazole | 3(0.8) | 6(3) | 2(1.2) | 11(1.5) |
| **J01DC Second-generation cephalosporins, n (%)** | | | | |
| Cefuroxime | 2(0.5) | 1(0.5) | 8(4.6) | 11(1.5) |

| | | | | |
|---|---|---|---|---|
| **J01EE Combinations of sulphonamides and trimethoprim, incl. Derivatives, n (%)** | | | | |
| Sulfamethoxazole and trimethoprim | 4(1.1) | 4(2) | 2(1.2) | 10(1.4) |
| **J01XX Other antibacterials, n (%)** | | | | |
| Linezolid | 2(0.5) | 3(1.5) | 0(0) | 5(0.7) |
| **J01AA Tetracyclines, n (%)** | | | | |
| Doxycycline | 1(0.3) | 0(0) | 0(0) | 1(0.1) |
| Minocycline | 0(0) | 2(1) | 0(0) | 2(0.3) |
| Tigecycline | 0(0) | 2(1) | 0(0) | 2(0.3) |
| **J01XB Polymyxins, n (%)** | | | | |
| Colistin | 0(0) | 5(2.5) | 0(0) | 5(0.7) |
| **J01XE Nitrofuran derivatives, n (%)** | | | | |
| Nitrofurantoin | 3(0.8) | 1(0.5) | 0(0) | 4(0.5) |
| **J01CF Dicloxacillin, n (%)** | | | | |
| Dicloxacillin | 1(0.3) | 1(0.5) | 1(0.6) | 3(0.4) |
| **J02AX Other antimycotics for systemic use, n (%)** | | | | |
| Caspofungin | 0(0) | 2(1) | 0(0) | 2(0.3) |
| **A07AA Antibiotics, n (%)** | | | | |
| Rifaximin | 0(0) | 0(0) | 1(0.6) | 1(0.1) |
| **J01CE Beta-lactamase sensitive penicillin, n (%)** | | | | |
| Benzylpenicillin | 1(0.3) | 0(0) | 0(0) | 1(0.1) |

Data are expressed as frequencies and percentages (n, %).
